# Systemic Design and Evaluation of Ticagrelor-Loaded Nanostructured Lipid Carriers for Enhancing Bioavailability and Antiplatelet Activity

**DOI:** 10.3390/pharmaceutics11050222

**Published:** 2019-05-08

**Authors:** Gi-Ho Son, Young-Guk Na, Hyun Wook Huh, Miao Wang, Min-Ki Kim, Min-Gu Han, Jin-Ju Byeon, Hong-Ki Lee, Cheong-Weon Cho

**Affiliations:** 1 College of Pharmacy and Institute of Drug Research and Development, Chungnam National University, 99, Daehak-ro, Yuseong-gu, Daejeon 34134, Korea; kyk2576@naver.com (G.-H.S.); youngguk@cnu.ac.kr (Y.-G.N.); wmclare@163.com (M.W.); hhw3573@nate.com (H.W.H.); zkzkang@naver.com (M.-K.K.); linuxfalcon@naver.com (M.-G.H.); jinju.byeon.cnu@gmail.com (J.-J.B.); 2 Present affiliation: Korea United Pharmaceutical Co. Ltd., 25-23, Nojangongdan-gil. Jeondong-myeon, Sejong 30011, Korea

**Keywords:** ticagrelor, nanostructured lipid carrier, design of experiments, Box–Behnken design, bioavailability, antiplatelet activity

## Abstract

Ticagrelor (TGL), a P2Y_12_ receptor antagonist, is classified as biopharmaceutics classification system (BCS) class IV drug due to its poor solubility and permeability, resulting in low oral bioavailability. Nanostructured lipid carriers (NLC) are an efficient delivery system for the improvement of bioavailability of BCS class IV drugs. Hence, we prepared TGL-loaded NLC (TGL-NLC) to enhance the oral bioavailability and antiplatelet activity of TGL with a systemic design approach. The optimized TGL-NLC with Box–Behnken design showed a small particle size of 87.6 nm and high encapsulation efficiency of 92.1%. Scanning electron microscope (SEM), differential scanning calorimetry (DSC), and powder X-ray diffraction (PXRD) were performed to investigate the characteristics of TGL-NLC. Furthermore, TGL-NLC exhibited biocompatible cytotoxicity against Caco-2 cells. Cellular uptake of TGL-NLC was 1.56-fold higher than that of raw TGL on Caco-2 cells. In pharmacokinetic study, the oral bioavailability of TGL-NLC was 254.99% higher than that of raw TGL. In addition, pharmacodynamic study demonstrated that the antiplatelet activity of TGL-NLC was superior to that of raw TGL, based on enhanced bioavailability of TGL-NLC. These results suggest that TGL-NLC can be applied for efficient oral absorption and antiplatelet activity of TGL.

## 1. Introduction

Ticagrelor (TGL), a cyclopentyl-triazolo-pyrimidine, is a new class of noncompetitive, direct-acting P2Y_12_ inhibitor that reversibly binds to the P2Y_12_ receptor [1]. It does not require metabolic activation in contrast to thienopyridines, like as clopidogrel and prasugrel, although the active metabolite, AR-C124910XX, attributed to its antiplatelet effects (approximately 30–40%) [1]. In the PLATO (Platelet Inhibition and Patient Outcomes) study, TGL showed a significant reduction of the incidence of cardiovascular death, myocardial infarction, and stroke in patients with acute coronary syndromes (ACS), compared to clopidogrel [2]. While TGL achieves rapid (approximately 2 h) and almost complete (approximately >90% of antiplatelet activity) antiplatelet activity that is more potent than that of clopidogrel, its time of action is significantly shorter as compared with prasugrel [3].

However, TGL is defined as “low solubility” under the BCS Class and is classified as BCS Class IV drug with poor permeability [4]. It has been reported that TGL has a low oral bioavailability of ≈36% and could be delivered directly from the liver to the bile [5]. Therefore, the trial of pharmaceutical development aims to improve oral bioavailability without the decrease of clinical effect.

In the last decade, nanotechnology is the fastest-growing strategy for increasing the solubility and permeability of BCS Class IV drug. In addition, nanomaterials produced by nanotechnology could avoid the immune response and penetrate multiple biological barriers [6].

Nanostructured lipid carrier (NLC), lipid-based nanoparticles, has been introduced as a new pharmaceutical delivery system [7]. NLC has certain advantages such as simple manufacturing process without organic solvents and easy scale up (e.g., high pressure homogenization). Moreover, NLC contains both solid and liquid lipids to form unstructured matrices, thus NLC improves drug loading capacity and reduces drug expulsion during storage [6]. Currently, the formulations such as solid dispersion and self-microemulsifying drug delivery systems have been developed to increase the bioavailability of TGL, but NLC formulations containing TGL have not yet been developed [8,9]. Therefore, the development of NLC is required to increase the bioavailability and the efficacy of TGL.

Optimization of the formulation or process is important to improve the regularity of the formulation during the development of the formulation. Systemic design is; therefore, used extensively to develop the formulation [10,11,12]. The application of statistical experimental designs, such as design of experiments, would be useful in understanding the relationship between factors and responses in a formulation. Herein, a Box–Behnken design was used to optimize TGL-NLC, which is useful for establishing quadratic or cubic response surfaces and constructing second order polynomial models [13].

The aim of the study was to develop TGL-loaded NLC through design of experiments. In addition, characteristics, in vitro release profile, cytotoxicity, and cellular uptake of TGL-NLC were evaluated. Pharmacokinetic and pharmacodynamic studies confirmed enhanced bioavailability and antiplatelet activity of TGL-NLC in rats.

## 2. Materials and Methods

### 2.1. Chemicals and Reagents

TGL was a gift from the Korea United Pharm Inc. (Seoul, Korea). High-performance liquid chromatography (HPLC) grade acetonitrile and methanol were used and purchased from JT Baker (Phillipsburg, NJ, USA). Capmul MCM (Glyceryl Caprylate/Caprate) was purchased from Abitec Corporation (Columbus, OH, USA). Stearic acid, palmitic acid, and myristic acid were obtained from Daejung Chemical (Cheongwon, Korea). Tween 80 and 20, Span 80 and 85, olive oil, oleic acid, and glyceryl monostearate were provided by Samchun Chemical (Pyungtaek, Korea). Gelucire 44/14, 50/13, 33/01 and 43/01, Capryol PGMC and 90, Compritol 888 ATO, Precirol ATO 5, Labrafac CC, Peceol, Labrafil M 1944 CS, Lauroglycol FCC, Lipophile WL 1349, Labrasol, Cremophor EL were purchased from Gattefossé (Saint-Priest, Cedex, France). Solutol HS-15, Poloxamer 188 and 407 were purchased from BASF (Ludwigshafen, Germany). Coumarin-6 (C6) and 3-(4,5-dimethylthiazol-2-yl)-2,5-diphenyl tetrazolium bromide (MTT) were purchased from Sigma-Aldrich (St. Louis, MO, USA). 4′,6-diamidino-2-phenylidone (DAPI) was purchased from Thermo Fisher Scientific (Waltham, MA, USA). All the reagents were of analytical grade and obtained from commercial sources.

### 2.2. HPLC Analysis

The concentration of TGL was determined by an HPLC method previously described with slight modification [8]. Agilent 1100 HPLC system (Agilent Technology, Santa Clara, CA, USA) with UV detector was used for the HPLC analysis. HPLC analysis was performed using a C18 analytical column (4.6 × 250 mm, 5 μm; Xterra RP18, Waters, Milford, MA, USA) maintained at 40 °C. The optimized mobile phase consisted of acetonitrile and buffer (50 mM ammonium acetate adjusted to pH 8.2 using 6 M ammonium hydroxide, 58:42%, *v*/*v*). The flow rate was 1.0 mL/min, and the injection volume was 20 µL. UV wavelength was set at 254 nm.

### 2.3. Solubility Study

The solubility of TGL to different solid lipids was evaluated by semi-quantitative method [14]. Briefly, 1 g of solid lipid was placed into a glass vial at a higher temperature than the melting point of solid lipids in water bath. Each 1 mg of TGL was added until the saturation point was reached. To evaluate the saturation solubility of TGL in different liquid lipids, an excess amount of drug was immersed in a 1 g of liquid lipid. The samples were mixed using a rotator for 72 h at room temperature (10 rpm, CUTE MIXER CM-1000, EYELA, Tokyo, Japan). Then, they were centrifuged at 15,000× *g* for 10 min (Gyro 1730 MR; Gyrozen, Daejeon, Korea). The supernatant was collected and diluted with 2-propanol. Diluents were analyzed by HPLC, and all experiments were triplicated. The saturation solubility of TGL in various 1 *w*/*v*% surfactants was evaluated using the described method above.

### 2.4. Preparation of TGL-NLC

Hot melt emulsification ultrasonication method was used to prepare the TGL-loaded NLC (TGL-NLC) [15]. Various ratios of glycerol monostearate and Capmul MCM were mixed, and they were melted at 70 °C in water bath. Then, 20 mg of TGL was added to form the homogenous and clear lipid phase. The aqueous phase (surfactants, Tween 80 and Poloxamer 188 with a 1:1 ratio in distilled water) was prepared under the same temperature as that of the lipid phase. The aqueous phase (10 mL) was added slowly to the lipid phase, and then the mixture was homogenized at 15,000× *g* for 2 min to obtain coarse emulsion (T 25 digital ULTRA-TURRAX^®^, IKA, Wilmington, NC, USA). They were sonicated with 50% amplitude for 5 min using an ultrasonicator (Vibra-Cell, Sonics & Material Inc., Newtown, CT, USA). Resulting dispersions (TGL-NLC) were cooled at 4 °C, and they were freeze-dried using lyophilizer (FD-1000, EYELA, Tokyo, Japan). Blank-NLC was prepared in the described method above without TGL.

### 2.5. Optimization of TGL-NLC

Based on the results of preliminary experiments, the TGL-NLC was optimized by Box–Behnken design with three factors and three responses (Table 1). The design of experiments and statistical analysis was conducted by Design Expert^®^ 11 software (Stat-Ease Inc, Minneapolis, MN, USA). Total lipid amount (X_1_), a ratio of liquid lipid/total lipid (X_2_), and percentage of surfactant (X_3_) were chosen as factors. In addition, particle size (Y_1_), polydispersity index (Y_2_), and encapsulation efficiency (Y_3_) of TGL-NLC were selected as responses to optimize the TGL-NLC. Seventeen of the designed experiments were conducted, and the resulting responses were fitted to linear, cubic, quadratic, special cubic, or quadratic polynomial models. To optimize the fitting model for each response, various statistical parameters, such as sequential p-values, lack of fit, squared correlation coefficient (R^2^), adjusted R^2^, and adequate precision were considered by comparing various statistical parameters provided by analyses of variance (ANOVA). After fitting the statistical model, the desirability value according to the goal of responses was obtained by numerical optimization and the TGL-NLC with the highest desirability value was prepared as the selected composition. A recovery test was performed to compare the error between the predicted and actual values.

#### 2.5.1. Particle size (Y_1_) and Polydispersity Index (Y_2_)

Physicochemical properties, including particle size and polydispersity index of TGL-NLC, were evaluated using electrophoretic light scattering analyzer (ELS-8000; Otsuka Electronics, Osaka, Japan). Briefly, the samples were sonicated to obtain an appropriate scattering intensity. The number of measurements was set at 50 times, and the average particle size and polydispersity index were measured. Measurement of the particle size and polydispersity index was conducted in triplication.

#### 2.5.2. Encapsulation Efficiency (Y_3_)

The encapsulation efficiency of TGL-NLC was evaluated by the ultrafiltration method [16]. Briefly, the cooled TGL-NLC dispersion before lyophilization was ultra-centrifugated with a centrifuge tube (MWCO 10 kDa, Amicon Ultra; Millipore, Billerica, MA, USA) for 20 min at 15,000g at 4 °C. The filtrate was diluted with acetonitrile to dissolve the free drug and the sample was analyzed by HPLC. The amount of free drug was designated as the amount of free TGL. The encapsulation efficiency was calculated by following equation: Encapsulation efficiency (%) = 100 × (total amount of TGL − amount of free TGL)/total amount of TGL.

### 2.6. Characterization of Optimized TGL-NLC

The particle size of TGL-NLC was performed using electrophoretic light scattering analyzer as described in Section 2.5.1. The morphology of lyophilized TGL-NLC was evaluated using a scanning electron microscope (SEM). Briefly, the fixed samples on an SEM-stub were coated with a thin layer of platinum in a vacuum. Then, TGL-NLC was installed in a cold type field emission (FE)-SEM (S-4800; Hitachi High-Technologies, Tokyo, Japan), and the morphology was observed.

Differential scanning calorimetry (DSC) analysis was conducted to evaluate the changes of thermal characteristics for TGL and lipid matrix using a thermal analyzer (DSC N-650, Scinco, Seoul, Korea). Two mg of samples were placed in an aluminum pan, and heated from 30 to 200 °C. The DSC thermogram was recorded with a heating rate of 10 °C/min under nitrogen flow.

For the evaluation of crystallinity of TGL-NLC, powder X-ray diffraction (PXRD) analysis was performed using a D/Max-2200 Ultima/PC (Rigaku Corporation, Tokyo, Japan) with Ni filtered Cu-Kα radiation (40 kV and 40 mA). Samples were scanned with the range of scan 2θ from 5° to 60° at room temperature, and PXRD patterns were recorded. Step size of 0.02°/s was set for continuous scan mode.

In vitro release of TGL was measured to determine that the TGL-NLC was absorbed in the form of NLC without degradation in the acidic environment. In vitro TGL release profile, in pH 1.2 medium containing 1 *w*/*v*% Tween 80, was conducted using a modified dialysis bag membrane diffusion technique [17]. Briefly, TGL-NLC was weighted and added into the pre-soaked cellulose membrane dialysis bag (25 kDa; Membra-Cel, Viskase, Inc., Chicago, IL, USA) with 2 mL of the medium. They were immersed in 100 mL of medium at 37 ± 0.5 °C under stirring (150 rpm). Aliquots of medium were taken at 0.25, 0.5, 1, 2, 4, 8, 12, 24, 48, and 72 h. Aliquots of fresh medium were added at sampling times. Samples were injected into HPLC, and the concentration of TGL was determined.

### 2.7. Cell Studies

#### 2.7.1. Cell Culture

Caco-2 cell line (human epithelial colorectal adenocarcinoma cell origin from human colon, KCLB No. 30037.1) was supplied from Korean Cell Line Bank (KCLB; Seoul, Korea). The cells were incubated in DMEM (10% heat-inactivated FBS, 100 units/mL of penicillin and 100 μg/mL of streptomycin) at 37 °C using an incubator (5% CO_2_ atmosphere). The cell was sub-cultured on every alternate day. Before the experiment, the cell was trypsinized with 0.25% trypsin-EDTA solution at 80% confluence.

#### 2.7.2. Cytotoxicity Study

The cytotoxicity of TGL and TGL-NLC was evaluated using the MTT assay. Briefly, 5 × 10^4^ cells of Caco-2 cell were seeded into plates, and they were incubated at 37 °C under 5% CO_2_ atmosphere for 24 h. TGL, blank-NLC, and TGL-NLC were diluted with 1% dimethyl sulfoxide in culture media to prepare various concentrations of TGL, ranging from 0.1 to 100 µg/mL. Blank-NLC was diluted to correspond to the lipid concentration of TGL-NLC. Then, TGL, blank-NLC, and TGL-NLC at an equivalent dose of TGL were treated to Caco-2 cell. After 24 h of incubation at 37 °C, MTT solution (30 µL) was added, and they were incubated for 3 h at 37 °C. After the culture media was completely removed, 200 μL of dimethyl sulfoxide was added to each well for dissolving transformed formazan crystals. The absorbance was measured at 560 nm using a microplate reader (Sunrise; Tecan Group Ltd., Mannedorf, Switzerland).

#### 2.7.3. Cellular Uptake Study

For the cellular uptake study of formulations, Caco-2 cells were seeded into 96-well plates at a density of 1 × 10^6^ cells per well and incubated for 24 h at 37 °C. Each well was treated with raw TGL and TGL-NLC at an equivalent concentration of 10 and 20 μg/mL of TGL, respectively. After 4 h of incubation, plates were washed 3 times with cold PBS, and cells were lysed by adding 0.5 mL of lysis buffer (1 *w*/*v*% Triton X-100 solution). TGL in cell lysis was extracted by adding acetonitrile. The concentration of TGL was determined by HPLC. Degree of cellular uptake of TGL was normalized with the amount of protein using BCA assay.

In addition, fluorescence observation was applied to visualize the cellular uptake of TGL-NLC in Caco-2 cells. For fluorescence staining, 1 mg of C6 was added in the preparation of TGL-NLC (C6-TGL-NLC). Caco-2 cells were seeded into 24-well plates at density of 1 × 10^4^ cells per well and incubated for 24 h at 37 °C. C6 solution and C6-TGL-NLC corresponding to 500 ng/mL C6 were diluted with 1% dimethyl sulfoxide in culture media and treated to each well. After 4 h incubation, the cells were washed 3 times with cold PBS and fixed with 4% formaldehyde solution for 10 min. After that, the nuclei of cells were stained with 300 nM DAPI solution for 5 min and washed twice with cold PBS. The stained cells were observed using EVOS^TM^ M5000 fluorescence microscopy (Thermo Fisher Scientific, Waltham, MA, USA). Control was the cells incubated only with culture media.

### 2.8. Pharmacokinetic Study

#### 2.8.1. Animal Study

All experiments were performed according to the guidelines of the Animal Care Commission of Chungnam National University (Daejeon, Korea). This study was approved by Chungnam National University Institutional Animal Care Committee (Protocol no. CNU-00911). Male Sprague Dawley rats (aged 7–8 weeks, body weight of 250–350 g) were obtained from Nara Biotech (Seoul, Korea), and housed at 22 °C for 2 weeks to adapt them to the environment.

Eighteen rats were randomly assigned to two groups (nine animals for each group). The raw TGC and TGC-NLC corresponding to 10 mg/kg of TGL were administrated to each group with oral gavage, respectively. The samples were dispersed to 0.5 *w*/*v*% carboxymethylcellulose solution prior to the administration. After the oral administration, blood was taken from the left jugular vein at 0, 0.17, 0.34, 0.68, 1, 1.5, 2, 4, 6, 8, 12, and 24 h. Blood was centrifuged at 15,000 × g for 10 min at 4 °C. Plasma was collected and stored immediately at −20 °C. The samples were analyzed with LC-MS/MS.

#### 2.8.2. LC-MS/MS analysis of TGL

Plasma concentration of TGL was determined by LC-MS/MS. Briefly, 20 µL of plasma sample was transferred to a 1.5 mL polypropylene tube. Four µL of DMSO and 100 µL of internal standard solution (verapamil, 200 ng/mL in acetonitrile) were added to the tube. They were vortexed and shaken for 5 min at room temperature. Then, they were centrifuged at 15,000× *g* for 10 min. Seventy µL of supernatant was transferred and diluted with distilled water (140 µL). The mixture was vortexed, and 10 µL of the mixture was injected into the LC-MS/MS. Data acquisition was performed with a TripleTOF™ 5600 System (AB SCIEX, Foster City, CA, USA) fitted with a Nanospray III source (AB SCIEX, Foster City, CA, USA) and a pulled quartz tip as the emitter (New Objectives, Woburn, MA). A C18 RP-MS column (50 × 4.6 mm, 2.6 μm; Accucore^TM^, Thermo Fisher Scientific, Waltham, MA, USA) was used, and the mobile phase was consisted of A (0.1% formic acid in distilled water) and B (0.1% formic acid in acetonitrile) with a gradient elution as follows: 0–0.5 min, hold at 10% B; 0.5–1 min, linear gradient from 10% to 25% B; 1–1.5 min, hold at 95% B, 1.5–1.6 min, linear gradient from 95% to 10%; 1.6–3.0 min, hold at 95% B at a flow rate of 0.4 mL/min. The positive mode was used to record the spectra of LC-MS/MS. The product ion of TGL (*m*/*z* 523.1) was selected with *m*/*z* 455 and that of verapamil (*m*/*z* 455.3) was *m*/*z* 165.1, respectively. The temperature of ion source and the curtain gas flow were set as 500 °C and 33 L/min, respectively. The ion spray was 4.5 kV. In the case of TGL. The optimized declustering potential and collision energy was 100 and 40 V for both TGL and IS, respectively. The concentration range of calibration curve was 3–2200 ng/mL for TGL and R^2^ value was >0.99.

#### 2.8.3. Pharmacokinetic Data Analysis

After the administration of TGL and TGL-NLC, pharmacokinetic parameters were determined by a non-compartmental model fitting using Phoenix WinNonlin 5.3.1 (Certara, Princeton, NJ, USA). Maximum concentration (C_max_) of TGL in plasma and the time to reach maximal concentration (T_max_) were determined from the data. Elimination half-life (T_1/2_) was calculated by ln 2/elimination rate constant. The area under the plasma concentration vs. time curve (AUC_0–∞_) was estimated using a linear trapezoidal rule. The oral relative bioavailability (RBA) of the TGL-NLC was calculated as: RBA (%) = 100 × AUC_0–∞_ of TGL-NLC/ AUC_0–∞_ of TGL.

### 2.9. Pharmacodynamic Study

For the evaluation of antiplatelet activity of TGL, light transmission aggregometry (LTA) method was used [8,18]. Whole blood was collected from the jugular vein at 0, 1, 4, 8, and 24 h after oral administrations of TGL and TGL-NLC corresponding to 10 mg/kg of TGL. Blood anti-coagulated with 3.2% sodium citrate was centrifuged at 220× *g* for 15 min at room temperature, and the supernatant (platelet-rich plasma, PRP) was collected. After collecting PRP, the residual blood was centrifuged at 2500× *g* for 10 min at room temperature to collect platelet-poor plasma (PPP).

To evaluate the ex vivo antiplatelet activities, an aggregometer (CHRONO-LOG^®^ Model 700, PA, USA) was used. Before the experiment, the number of platelets in the PRP was measured and adjusted to 5 × 10^7^ platelets/mL by diluting with PPP using a hematology analyzer (ADVIA 2120i; Siemens Healthineers, Forchheim, Germany). Two hundred and forty µL of adjusted PRP was warmed for 1 min at 37 °C under stirring. After the warming of PRP, 10 µL of ADP (20 μM) was added to induce the platelet aggregation. Degree of platelet aggregation was measured and recorded for 10 min after addition of ADP. The area under the curve (AUC) of aggregometry was measured by aggregometer and the inhibition of platelet activity (IPA) was calculated by following equation: IPA (%) = 100 × {1 − (AUC of sample/AUC of blank)}. Where the AUC of blank is the AUC at 0 h and the AUC of sample is the AUC at each time-point after single oral administration of TGL-NLC, respectively. The area under the inhibitory curves of platelet aggregation (AUIC_0–24_) was estimated using a linear trapezoidal rule.

### 2.10. Statistical Analysis

All values in this study were presented as the mean ± SD except for pharmacodynamic data. Pharmacodynamic data were expressed as mean ± SEM. The significant difference was evaluated with Prism 8 (GraphPad Software, CA, USA) according to student t-test (*p* < 0.05) and one-way analysis of variance (ANOVA).

## 3. Results

### 3.1. Optimization and Characterization of TGL-NLC

#### 3.1.1. Solubility Study for TGL-NLC

**Figure 1:**
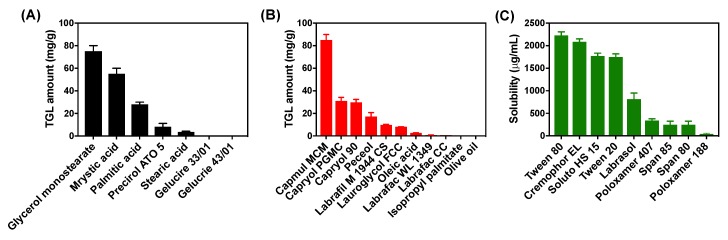
The solubility of ticagrelor (TGL) in (**A**) solid lipids, (**B**) liquid lipids, and (**C**) 1 *w*/*v*% surfactant solutions. Data are expressed as the mean ± SD (n = 3).

The solubility of TGL in the lipid matrix is a major factor affecting the encapsulation efficiency of TGL in NLC. Moreover, the type of lipids and surfactants constituting NLC has a large influence on the particle size of NLC [19]. Thus, the solubility studies of TGL for solid lipids, liquid lipids, and surfactants were carried out prior to the preparation of TGL-NLC. The solubility of TGL in various solid lipids is shown in Figure 1A. Glycerol monostearate showed the highest solubility (75.3 ± 5.3 mg/g). Glycerol monostearate tends to form amorphous forms rather than crystalline forms by incorporation with drugs. In contrast, the solubility of TGL in saturated fatty acids (stearic acid, palmitic acid, myristic acid) was lower than that of glycerol monostearate due to its high crystallinity. These lipids are inherently high crystalline and can incorporate a small amount of drug. However, the high solubility of drug on lipids is a prerequisite for preparing NLC with high drug loading, which ensures appropriate incorporation of drug and lipid in vivo [20]. Thus, we chose glycerol monostearate as a solid lipid to prepare TGL-NLC.

The solubility of TGL in liquid lipids iss shown in Figure 1B. TGL exhibited low solubility in long-chain unmodified oils (2.7 ± 0.1 mg/g and 0.1 ± 0.0 mg/g for oleic acid and olive oil, respectively), whereas, TGL showed high solubility in semisynthetic modified oils (i.e., Capmul MCM, Capryol PGMC, and Capryol 90). Among the screened liquid lipids, Capmul MCM exhibited the highest solubility of TGL (85.0 ± 4.9 mg/g). Hence, Capmul MCM was selected as a suitable liquid lipid.

Surfactants play a crucial role in the formation and stabilization of NLC [21]. The solubility of TGL showed the lowest solubility in Poloxamer 188 (40.1 ± 19.4 µg/mL) and, conversely, the highest solubility in Tween 80 (2228.3 ± 79.5 µg/mL) (Figure 1C). Sharma et al. reported that Poloxamer 188, which exhibits the lowest solubility, can be used to stabilize the surface of NLC [22]. Tween 80 was also used as a surfactant for incorporating TGL into TGL-NLC due to hydrophilic polyethylene oxide chain and lipophilic fatty acid group [23]. Additionally, Patel et al. suggested that using Poloxamer 188 (1%), a steric stabilizer, with Tween 80 (2%) as a surfactant in the manufacture of NLC could provide a stable NLC with a particle size below 100 nm [24]. Thus, a combination of Poloxamer 188 and Tween 80 (1:1 ratio) was used as a surfactant in our TGL-NLC preparation.

#### 3.1.2. Optimization of TGL-NLC

Box–Behnken design was used to optimize the TGL-NLC by fitting the response resulted from experiments with Design Expert^®^ 11. The design is desirable for the response surface methodology for optimizing formulation and process. Compared with central composite design, mixture design, and three-level factor design, the advantages of Box–Behnken design is lower cost and more time savings due to fewer experiments. Additionally, since there is no axial point in the design, all design points can belong to a safe operating area. The suitable fitting models for the responses are suggested through statistical analysis [25]. The particle size (Y_1_), polydispersity index (Y_2_), and encapsulation efficiency (Y_3_) of TGL were considered as crucial responses in evaluating the optimized TGL-NLC that exhibited good stability and improved bioavailability. The small Y_1_ value means that the NLC with small particle size can penetrate the intestinal membrane through intracellular and paracellular pathways, thereby enhancing the oral bioavailability of TGL [26]. The small Y_2_ value indicates that the particle size distribution of TGL-NLC is narrow and homogeneous [27]. Furthermore, the high Y_3_ means that a high amount of drug encapsulated in the NLC, and the highly encapsulated TGL can easily penetrate via intestinal membrane because of the lipophilicity of the NLC [28].

The statistical models of Y_1_, Y_2_, and Y_3_ responses were fitted as linear, quadratic, and linear models, respectively (Table 2). Statistical values such as sequential *p*-value, lack of fit, R^2^, adjusted R^2^, and adequate precision were evaluated to determine the suitability of the models [29]. In all the suggested models, the sequential p-values less than 0.05 indicated that the statistical hypothesis of the suggested model was significant at the 95% confidence level [30]. The lack of fit *p*-values of the models were more than 0.05, meaning that the suggested models were proper to evaluate the relationship between factor and response. [31]. The R^2^ and adjusted R^2^ values reflected how the variability of the suggested model was consistent with the actual values [32]. R^2^ values of Y_1_, Y_2_, and Y_3_ were more than 0.8, meaning that the models of the actual values were similar to the statistical models [33]. In addition, the difference between the R^2^ and adjusted R^2^ of all responses was less than 0.2, which could be interpreted as a good fit between the model of the actual values and the model of the predicted values [34].

**Figure 2:**
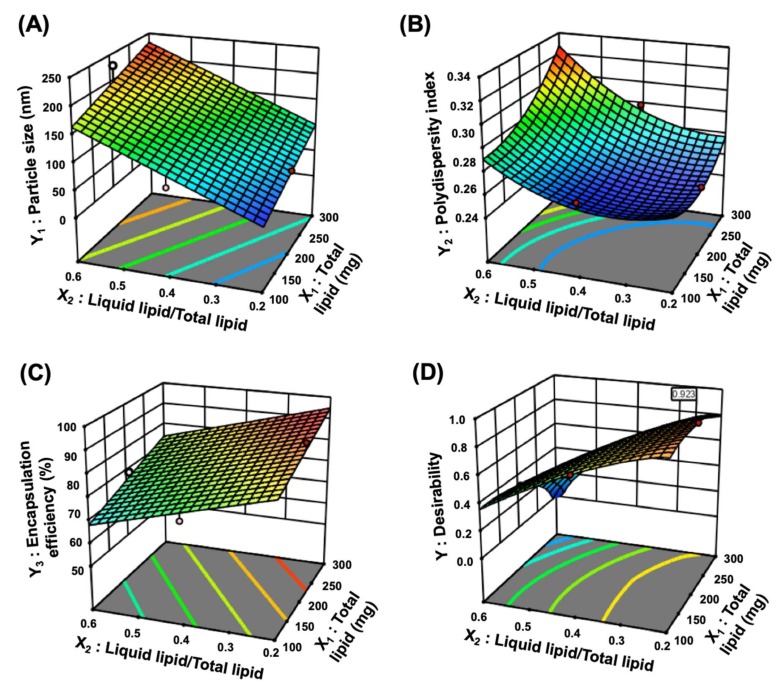
3D surface plots of (**A**) Y_1_: Particle size, (**B**) Y_2_: Polydispersity index, (**C**) Y_3_: Encapsulation efficiency, and (**D**) Y: Desirability value. The X_3_ (percentage of surfactant) was set to 1 *w*/*v*%.

Three-dimensional plots and coefficient equations were applied to investigate the relationships between each factor (Figure 2 and Table S1, supplementary data). The high percentage of X_3_ prevents the TGL from being encapsulated and lowers the Y_3_ value, so the value of X_3_ was set as 1 *w*/*v*%. Figure 2 and Table 3 show the statistical patterns and actual values of the responses. The ranges of Y_1_, Y_2_, and Y_3_ were from 76.1 to 151.2 nm, 0.279 to 0.361, and 74.26% to 95.14%, respectively. In Table S1 (supplementary data), a positive coefficient represents a synergistic effect, while a negative coefficient indicates an antagonistic effect [35]. In the case of Y_1_, the positive coefficients of X_1_ and X_2_ indicated an increase in particle size as each factor increased (Table S2, supplementary data). The increase in Y_1_ when increasing X_1_ may be due to increased viscosity of the o/w emulsion formed during the ultrasonication. Additionally, as X_1_ and X_2_ increased, the effect of synergy with the increase in Y_2_ was observed (Table S3, supplementary data). In the case of Y_3_, it increased as X_1_ increased and X_2_ decreased (Table S4, supplementary data). These results are due to an increase in the amount of drug that can be dissolved as the total lipid amount increased. In addition. when the ratio of liquid lipid to total lipid is increased, the liquid lipid is not sufficiently incorporated into the solid lipid, and then the incorporation of drug into lipid matrix is lowered by separation of liquid lipid to the outer water phase.

The optimal factors were determined using the desirability function reflecting all responses. The goals of Y_1_ and Y_2_ were set to be minimized and that of Y_3_ was set to be maximized. Figure 2D presents the desirability plot drawn considering the influence of factors on all responses. The optimal X_1_, X_2_, and X_3_ were 189.3 mg, 0.2, and 1%, respectively, and the desirability value of this formulation was 0.923. The predicted values and the actual values are listed in Table 4. To evaluate the accuracy of the predictions, the error between the predicted value and actual value of each response was calculated as an error percentage. The errors associated with Y_1_, Y_2_, and Y_3_ were 3.3%, 6.2%, and 1.1%, respectively. The percentage of less than 10% indicated that the optimization of TGL-NLC was successfully achieved [36].

#### 3.1.3. Characterization of Optimized TGL-NLC

**Figure 3:**
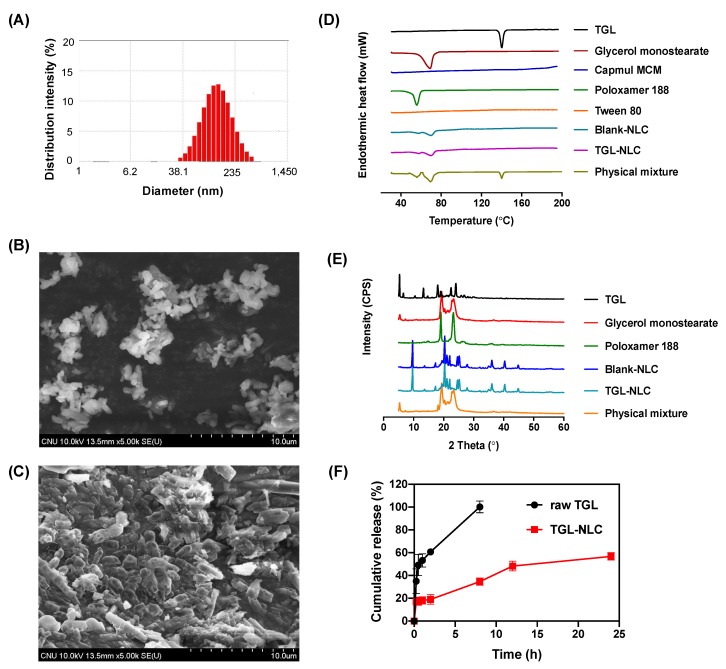
(**A**) Particle size distribution of TGL-NLC measured with electrophoretic light scattering (ELS) analyzer. The SEM image of (**B**) raw TGL and (**C**) TGL-NLC. (**D**) DSC thermograms of raw TGL, glycerol monostearate, Capmul MCM, poloxamer 188, Tween 80, blank-NLC, TGL-NLC, and physical mixture. (**E**) PXRD patterns of raw TGL, glycerol monostearate, poloxamer 188, blank-NLC, TGL-NLC, and physical mixture. (**F**) In vitro TGL release profiles of raw TGL and TGL-NLC in pH 1.2 medium with 1 *w*/*v*% Tween 80. Data are expressed as the mean ± SD (n = 3). Abbreviation: SEM, scanning electron microscopy; DSC, differential scanning calorimetry; PXRD, powder X-ray diffraction; blank-NLC, nanostructured lipid carriers without ticagrelor.

The TGL-NLC showed a particle size of 87.6 ± 6.6 nm as measured by electrophoretic light scattering analyzer (Figure 3A). A SEM was used to observe the morphologies of raw TGL and TGL-NLC. The raw TGL showed cylindrically shaped crystals depicting the crystalline nature of the drug (Figure 3B). However, as shown in Figure 3C, TGL-NLC showed a smooth, rounded surface uniformly. These images indicated that TGL was successfully incorporated into NLC.

A DSC was conducted to investigate the crystallinity of TGL in the TGL-NLC. Figure 3D showed the DSC thermograms of raw TGL, glycerol monostearate, Capmul MCM, poloxamer 188, Tween 80, blank-NLC, TGL-NLC, and physical mixture. An endothermic peak was observed at 138 °C, which corresponds to the melting point of TGL, which reflects its crystallinity [9]. Moreover, the thermal peaks of glycerol monostearate and poloxamer 188 were shown at 61 and 54 °C, respectively. In the physical mixture, the sharp endothermic peaks of TGL, glycerol monostearate, and poloxamer 188 were observed, while no peak of TGL was detected in TGL-NLC. In addition, the intensity of glycerol monostearate and poloxamer 188 was reduced in TGL-NLC.

Figure 3E illustrated the PXRD spectra of TGL, glycerol monostearate, poloxamer 188, blank-NLC, TGL-NLC, and physical mixture. A number of peaks indicating crystallinity were detected in the raw TGL. In TGL-NLC, the peak intensity of glycerol monostearate and poloxamer 188 was remarkably reduced and the peaks exhibiting another crystallinity were found by the preparation of NLC. In addition, no intense peaks meaning the crystallinity of TGL were detected and the peaks were similar to those of blank-NLC. However, in the case of physical mixture, the major peaks of glycerol monostearate and poloxamer 188 were detected. These results indicate that TGL is completely incorporated into the lipid matrix in an amorphous and/or solubilized state in TGL-NLC.

In vitro release tests were performed to investigate the TGL release profile on pH 1.2 medium containing 1 *w*/*v*% Tween 80 with a dialysis bag. Figure 3F represented the cumulative release of raw TGL and TGL-NLC for 24 h. The raw TGL released 90% of TGL within 8 h, while the TGL-NLC showed a sustained drug release profile, with a burst release (≈17%) within 0.25 h, followed by a prolonged release for up to 24 h (≈60%). The initial burst release of TGL from TGL-NLC may occur because the TGL attached to the surface of the TGL-NLC is immediately released to the medium [37]. Subsequent sustained release may be attributed to degradation and erosion of the drug-incorporated lipid matrix [38]. In addition, the profile supported that the TGL was substantially encapsulated within the lipid matrix. The sustained release profile may be due to by the use of glycerol monostearate, a major component of the lipid matrix, commonly known as a sustained release agent [39]. This result was supported by the previous study in which the release of NLC prepared with glycerol monostearate showed a slow release profile of ≈50% for 72 h [14]. These properties of TGL-NLC would be beneficial to prevent the drug degradation from the acidic environment and increase intestinal absorption in the form of NLC, which would increase lymphatic uptake and avoid CYP-mediated hepatic first-pass metabolism [40].

### 3.2. Cell Studies

#### 3.2.1. Cytotoxicity Study

**Figure 4:**
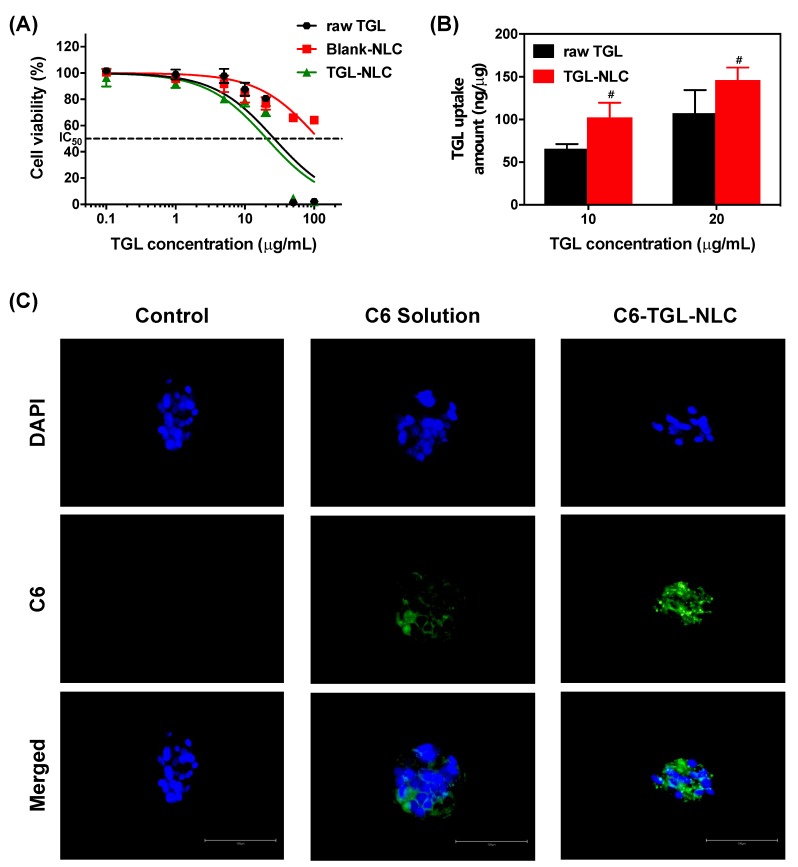
(**A**) Cytotoxicity of raw TGL, blank-NLC, and TGL-NLC against Caco-2 cells for 24 h. Data are expressed as the mean ± SD (n = 5). (**B**) Cellular uptake of TGL from raw TGL powder and TGL-NLC into Caco-2 cells incubated for 4 h. The TGL uptake amount was normalized by protein amount of cell lysates. Data are expressed as the mean ± SD (n = 4). ^#^ *p* < 0.05 vs. raw TGL at the same dose. (**C**) Cellular uptake study in Caco-2 cell lines observed with fluorescence microscope (incubated for 4 h with coumarin-6 (C6) solution and C6-TGL-NLC). Blue and green colors indicated DAPI staining and coumarin-6 (C6), respectively. The length of the white bar is 100 µm, which applies to all images including DAPI and C6 images. Abbreviation: DAPI, 4′,6-diamidino-2-phenylidone.

The cytotoxicity of raw TGL, blank-NLC, and TGL-NLC against Caco-2 cells was evaluated with MTT assay. Caco-2 cells are used as an in vitro intestinal model since they differentiate monolayers with tight junctions and transport systems [41]. Usually, the cell viability above 70% is generally considered as “no toxicity” [42]. Meanwhile, the cell viability of less than 50% is considered as “irritation” [43]. In this study, blank-NLC was prepared with the lipid concentration equivalent to that of TGL-NLC. Figure 4A exhibited cell viability of three formulations after 24 h incubation. The raw TGL and TGL-NLC produced the dose-dependent cytotoxicity. The TGL-NLC showed a similar degree of cytotoxicity on cell proliferation of Caco-2 cells with raw TGL. The IC_50_ of raw TGL and TGL-NLC were 26.54 and 20.72 μg/mL, respectively. However, in the case of blank-NLC, the cytotoxicity against Caco-2 cells was not found. These results suggested that the solid lipid, liquid lipid, and surfactant incorporated in NLC were not toxic to Caco-2 cells until the concentration of blank-NLC was ≈1.61 mg/mL (corresponding to 100 μg/mL of TGL), while the raw TGL and TGL-NLC exhibited significant cytotoxicity when the concentration of TGL was >12.5 μg/mL. Interestingly, the cytotoxicity was found to be related to the concentration of TGL, not the NLC formulation. The NLC formulation is highly biocompatible and can be used as a drug delivery vehicle for oral administration, and TGL-NLC may exhibit similar cytotoxicity in vitro to commercial TGL product (Brilinta^®^) according to the dose of drug.

#### 3.2.2. Cellular Uptake Study

To investigate the effect of TGL-NLC on the enhancement of TGL uptake into Caco-2 cells, in vitro cellular uptake study was performed on Caco-2 cells. The cells were treated with raw TGL and TGL-NLC corresponding to 10 and 20 μg/mL of TGL, respectively, for 4 h, and then intracellular TGL concentration was analyzed by HPLC. As shown in Figure 4B, the cellular uptakes of TGL-NLC were significantly increased by 1.56-fold and 1.36-fold compared to those of raw TGL at 10 and 20 μg/mL of TGL, respectively (*p* < 0.05). In addition, cell uptake by TGL-NLC at 10 μg/mL TGL (102.31 ng/µg) was similar to that of raw TGL at 20 μg/mL TGL (107.36 ng/µg). These results indicated that cellular uptake of TGL was enhanced by TGL-NLC.

Fluorescence microscopy was conducted to evaluate the cellular uptake of NLC in Caco-2 cells (Figure 4C). Interestingly, the fluorescence intensity of C6-TGL-NLC was stronger than that of the C6 solution. These results indicated that the cellular uptake or accumulation of C6-TGL-NLC in Caco-2 cells was enhanced compared to that of C6 solution. C6-TGL-NLCs allowed more C6 to uptake the cells than C6 solution due to their ability to provide more lipophilic, nanoscale, and occlusive effect. The large surface area by nanosized NLC promotes the contact between the drug and the cell membrane, thereby improving the cell uptake of drug. Furthermore, the synergistic mechanism between lipids and surfactants can enhance drug absorption by enhancing affinity for cell membranes with lipophilicity. Therefore, these results supported that TGL could be taken up into Caco-2 cells by incorporation into NLC.

### 3.3. Pharmacokinetic Study

**Figure 5:**
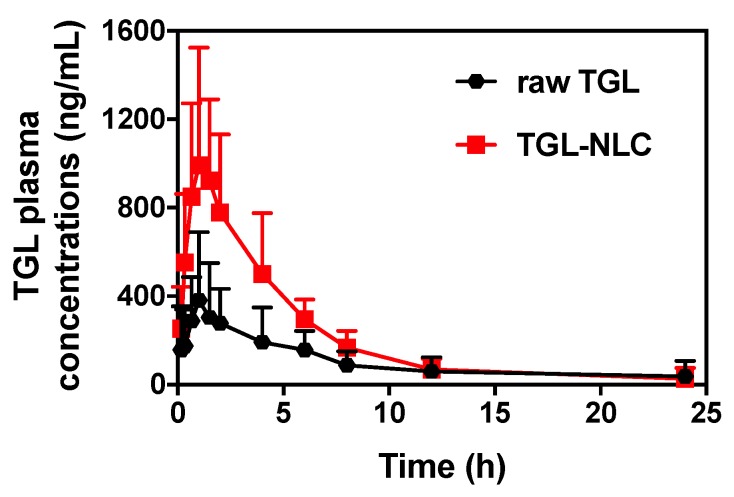
Plasma concentration-time curve of TGL in rats after single oral administration of raw TGL and TGL-NLC at a dose equivalent to 10 mg/kg of TGL. Data are expressed as the mean ± SD (n = 9).

To evaluate the pharmacokinetics of formulations, raw TGL and TGL-NLC corresponding to 10 mg/kg of TGL were orally administered to rats (Figure 5). The relevant pharmacokinetic parameters of each formulation are shown in Table 5. After oral administration, C_max_ and AUC_0–∞_ of TGL were significantly higher than those of raw TGL (*p* < 0.05). As expected, raw TGL showed lower C_max_ (461.75 ± 88.77 ng/mL) and AUC_0–∞_ (2103.01 ± 283.36 ng∙h/mL) than the C_max_ (1050.44 ± 170.14 ng/mL) and AUC_0–∞_ (5362.43 ± 808.51 ng∙h/mL) of TGL-NLC. In the case of T_max_, TGL-NLC showed slightly reduced time (1.20 ± 0.12 h) compared to that of raw TGL (2.65 ± 0.82 h). As a result, the RBA of TGL-NLC was 254.99% based on the results of AUC data. The enhanced oral bioavailability of TGL-NLC was related to the physical characteristic of TGL-NLC and results of cell studies, showing that the small-sized TGL-NLC (<100 nm) could be efficiently absorbed into the blood circulation through the gastrointestinal tract. These results might be due to the high cellular uptake in Caco-2 cells and improved affinity with Caco-2 cells, enhancing the permeability across the intestinal barrier [44]. Moreover, the NLC with small particle size could have a positive effect on intracellular transport and paracellular transport, thereby improving the intestinal absorption and preventing drug degradation by intestinal enzymes [45]. Additionally, the relatively small mass of TGL-NLC might increase the adhesion of TGL-NLC to the intestinal mucosa, thereby improving the intestinal absorption and the oral bioavailability [46].

### 3.4. Pharmacodynamic Study

**Figure 6:**
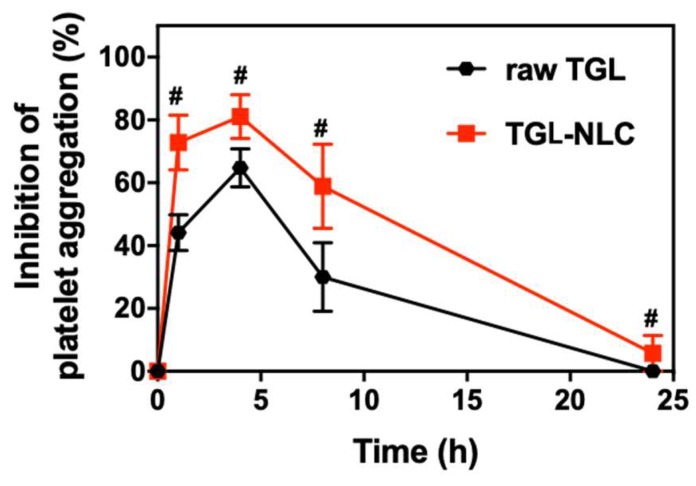
Ex vivo antiplatelet activities after single oral administration of raw TGL and TGL-NLC at a dose equivalent to 10 mg/kg of TGL. Data are expressed as the mean ± SEM (n = 6). ^#^ *p* < 0.05 vs. raw TGL at the same time-point.

Ex vivo antiplatelet activity was evaluated to investigate the platelet aggregation induced by 20 μM ADP after a single oral administration of raw TGL and TGL-NLC. Blood samples were collected at 1, 4, 8, and 24 h after administration. As shown in Figure 6, both raw TGL and TGL-NLC exhibited a time-course manner of IPA. The maximum level of IPA was reached at 4 h, and the antiplatelet activity lasted for 8 h. The IPA of TGL-NLC was superior to that of raw TGL during all experiment period. Moreover, the AUIC_0–24_ data presented that the level of antiplatelet activity of TGL-NLC was greater compared with that of raw TGL (Table 6). The AUIC_0–24_ of TGL-NLC was 1064.2 ± 121.5 %∙h, which was 1.73-fold higher than that of raw TGL (615.0 ± 91.9%∙h), indicating that significantly increased antiplatelet activity of TGL by oral administration of TGL-NLC (*p* < 0.05). These results showed that TGL-NLC not only enhanced the oral bioavailability but also increased antiplatelet activity by improving drug absorption, as shown in pharmacokinetic study.

## 4. Conclusions

TGL-NLC for enhancing oral bioavailability and antiplatelet activity of TGL was successfully optimized using the design of experiments. The optimized TGL-NLC exhibited small particle size, narrow size distribution, and high encapsulation efficiency. Moreover, the formulation showed a sustained release profile, low cytotoxicity, and enhanced cellular uptake on Caco-2 cells. In pharmacokinetic study, the oral bioavailability of TGL by TGL-NLC was 254.99% higher than that of raw TGL. Furthermore, the results from the ex vivo pharmacodynamic study demonstrated that TGL-NLC showed greater antiplatelet activity than raw TGL at the same dose. In conclusion, we successfully developed the TGL-NLC thorough systemic design and evaluated the formulation. The formulation could be a promising approach to TGL delivery with significantly enhanced oral bioavailability and antiplatelet activity. Further investigation of TGL-NLC compared to various formulation of TGL will be performed to select the appropriate formulation supporting future clinical applications. Additionally, it is necessary to study the stability of NLC for long-term storage. Therefore, the encapsulation efficiency, particle size, and impurities will be examined for 36 months.

## Figures and Tables

**Table 1:** Factors and responses used in Box–Behnken design.

**Factors**	**Range**	
	**Low Limit (mg)**	**High Limit (mg)**
X_1_: Total lipid amount	100	300
X_2_: Ratio of liquid lipid/total lipid	0.2	0.6
X_3_: Percentage of surfactant	1	3
**Responses**	**Goal**	
Y_1_: Particle size (nm)	Minimize	
Y_2_: Polydispersity index	Minimize	
Y_3_: Encapsulation efficiency (%)	Maximize	

**Table 2:** Statistical parameters for suggested models of responses.

Responses	Suggested Model	Model *p*-Value	Lack of Fit *p*-Value	R^2^	Adjusted R^2^	Adequate Precision
Y_1_: Particle size (nm)	Linear	<0.0001	0.7090	0.8570	0.8241	17.2218
Y_2_: Polydispersity index	Quadratic	0.0139	0.4403	0.8740	0.7119	7.8183
Y_3_: Encapsulation efficiency (%)	Linear	<0.0001	0.7622	0.8430	0.8068	15.0182

**Table 3:** Experimental design and actual responses of Box–Behnken design. Data are expressed as mean ± SD (n = 3).

Run	Factors			Responses		
	X_1_	X_2_	X_3_	Y_1_	Y_2_	Y_3_
	Total Lipid Amount (mg)	Ratio of Liquid Lipid/Total Lipid	Percentage of Surfactant (%)	Particle Size (nm)	Polydispersity Index	Encapsulation Efficiency (%)
1	200	0.6	1	151.2 ± 6.5	0.311 ± 0.031	84.25 ± 3.15
2	200	0.4	2	104.2 ± 3.5	0.303 ± 0.012	86.14 ± 1.42
3	200	0.4	2	114.2 ± 3.4	0.312 ± 0.031	85.32 ± 2.41
4	200	0.4	2	102.3 ± 8.1	0.317 ± 0.021	83.12 ± 3.36
5	100	0.4	3	84.2 ± 3.6	0.331 ± 0.024	78.26 ± 1.48
6	200	0.6	3	124.8 ± 5.7	0.277 ± 0.019	78.24 ± 2.85
7	300	0.2	2	104.8 ± 8.1	0.331 ± 0.027	95.12 ± 3.14
8	200	0.4	2	121.1 ± 7.1	0.342 ± 0.018	90.42 ± 1.45
9	100	0.6	2	132.1 ± 8.3	0.347 ± 0.014	74.26 ± 2.85
10	200	0.2	3	80.3 ± 3.4	0.36 ± 0.025	87.36 ± 1.64
11	300	0.4	1	115.2 ± 3.2	0.319 ± 0.037	91.34 ± 1.75
12	200	0.4	2	93.5 ± 2.7	0.308 ± 0.021	83.25 ± 2.34
13	100	0.4	1	91.5 ± 1.9	0.285 ± 0.033	82.64 ± 3.15
14	100	0.2	2	76.1 ± 3.1	0.361 ± 0.022	83.14 ± 1.48
15	300	0.4	3	124.3 ± 1.4	0.277 ± 0.027	81.26 ± 2.95
16	300	0.6	2	149.2 ± 2.5	0.36 ± 0.024	82.15 ± 3.48
17	200	0.2	1	88.1 ± 3.5	0.279 ± 0.018	95.14 ± 4.52

**Table 4:** Predicted and actual values of responses for optimized ticagrelor-loaded nanostructured lipid carriers (TGL-NLC). Actual values are expressed as mean ± SD (n = 3).

Optimized Factors	Responses	95 % CI * Low Predicted Value	Predicted Value	95% CI * High Predicted Value	Actual Value	Error Percentage (%)
X_1_: 189.3 mg	Y_1_: Particle size (nm)	74.4	85.8	97.3	87.6 ± 6.6	2.1
X_2_: 0.2	Y_2_: Polydispersity index	0.244	0.276	0.308	0.259 ± 0.013	6.2
X_3_: 1.0%	Y_3_: Encapsulation efficiency (%)	90.1	93.1	96.2	92.1 ± 3.1	1.1

* Abbreviation: CI, confidence interval.

**Table 5:** Pharmacokinetic parameters after single oral administration of raw TGL and TGL-NLC in rats. Data are expressed as the mean ± SD (n = 9). ^#^ *p* < 0.05 vs. raw TGL.

Pharmacokinetic Parameters	Samples	
	Raw TGL	TGL-NLC
T_max_ (h)	2.65 ± 0.82	1.20 ± 0.12
C_max_ (ng/mL)	461.75 ± 88.77	1050.44 ± 170.14 ^#^
AUC_0–∞_ (ng·h/mL)	2103.01 ± 283.36	5362.43 ± 808.51 ^#^
T_1/2_ (h)	3.46 ± 0.56	4.80 ± 1.21
RBA (%) vs. raw TGL		254.99

Abbreviation: T_max_, time to reach maximal concentration; C_max_, maximum concentration; AUC_0–∞_, area under the plasma concentration vs. time curve; T_1/2_, elimination half-life; RBA, oral relative bioavailability.

**Table 6:** Area under the inhibitory curves of platelet aggregation (AUIC) by administration of raw TGL and TGL-NLC in rats. Data are expressed as the mean ± SD (n = 6). ^#^ *p* < 0.05 vs. raw TGL.

Parameter	Samples	
	Raw TGL	TGL-NLC
AUIC_0–24_ (%⋅h)	615.0 ± 91.9	1064.2 ± 121.5 ^#^

## Supplementary Materials

The following are available online at https://www.mdpi.com/1999-4923/11/5/222/s1, Table S1: Coefficient equations of responses according to the level of factors, Table S2: Analysis of variance for model of particle size (Y_1_), Table S3: Analysis of variance for model of polydispersity index (Y_2_), Table S4: Analysis of variance for model of encapsulation efficiency (Y_3_).
