# Supplementary material for: Systemic Design and Evaluation of Ticagrelor-Loaded Nanostructured Lipid Carriers for Enhancing Bioavailability and Antiplatelet Activity
Source: Pharmaceutics. 2019 May 8;11(5):222. doi: 10.3390/pharmaceutics11050222 (PMC6572397; doi:10.3390/pharmaceutics11050222)
Supplement: Supplementary file 1 [file pharmaceutics-11-00222-s001.pdf]

pharmaceutics

MDPI

Article

Systemic Design and Evaluation of Ticagrelor-Loaded Nanostructured Lipid Carriers for Enhancing Bioavailability and Antiplatelet Activity

Gi-Ho Son [1,2,†], Young-Guk Na [1,†], Hyun Wook Huh [1], Miao Wang [1], Min-Ki Kim [1], Min-Gu Han [1], Jin-Ju Byeon [1], Hong-Ki Lee [1,*] and Cheong-Weon Cho [1,*]

1 College of Pharmacy and Institute of Drug Research and Development, Chungnam National University, 99, Daehak-ro, Yuseong-gu, Daejeon 34134, Korea; kyk2576@naver.com (G.-H.S.); youngguk@cnu.ac.kr (Y.-G.N.); wmclare@163.com (M.W.); hhw3573@nate.com (H.-W.H.); zkzkang@naver.com (M.-K.K.); linuxfalcon@naver.com (M.-G.H.); jinju.byeon.cnu@gmail.com (J.-J.B.)

2 Present affiliation: Korea United Pharmaceutical Co. Ltd., 25-23, Nojangongdan-gil. Jeondong-myeon, Sejong 30011, Korea

* Correspondence: chocw@cnu.ac.kr (C.-W.C.); dvmlhk@gmail.com (H.-K.L.); Tel.: +82-42-821-5934 (C.-W.C.); +82-42-821-7301 (H.-K.L.)

† These authors contributed equally to this work.

Received: 15 April 2019; Accepted: 6 May 2019; Published: 8 May 2019

1. Supplementary tables

Table 1. Coefficient equations of responses according to the level of factors.

Responses	Coefficient equations
Y_1	$109.24 + 13.70X_1 + 26.00X_2 - 4.05X_3$
Y_2	$0.3164 - 0.0046X_1 - 0.0045X_2 + 0.0064X_3 + 0.0107X_1X_2 - 0.0220X_1X_3 - 0.0287X_2X_3 + 0.0148X_1^2 + 0.0185X_2^2 - 0.0282X_3^2$
Y_3	$84.79 + 3.95X_1 - 5.23X_2 - 3.53X_3$

Table 2. Analysis of variance for model of particle size (Y_1).

Source	df	Mean Square	F-value	p-value
Model	3	2346.91	25.98	< 0.0001
X_1	1	1501.52	16.62	0.0013
X_2	1	5408.00	59.836	< 0.0001
X_3	1	131.22	1.45	0.2496
Residual	13	90.34		
Lack of fit	9	79.07	0.6833	0.7090
Pure error	4	115.70		
Total	16			

Note: df (degree of freedom); a large F-value implies a large impact on the modeling profile; a p-value of less than 0.05 means that it affects modeling profile.

Table 3. Analysis of variance for model of polydispersity index (Y_2).

Source	df	Mean Square	F-value	p-value
Model	9	0.0013	5.39	0.0185
X_1	1	0.0002	0.7038	0.4292
X_2	1	0.0002	0.6663	0.4412
X_3	1	0.0003	1.34	0.2855
X_1X_2	1	0.0005	1.90	0.2104
X_1X_3	1	0.0019	7.96	0.0257
X_2X_3	1	0.0033	13.60	0.0078
X_1^2	1	0.0009	3.79	0.0925
X_2^2	1	0.0014	5.96	0.0447
X_3^2	1	0.0033	13.77	0.0075
Residual	7	0.0002		
Lack of fit	3	0.0003	1.12	0.4403
Pure error	4	0.0002		
Total	16			

Note: df (degree of freedom); a large F-value implies a large impact on the modeling profile; a p-value of less than 0.05 means that it affects modeling profile.

Table 4. Analysis of variance for model of encapsulation efficiency (Y_3).

Source	df	Mean Square	F-value	p-value
Model	3	147.67	23.27	< 0.0001
X_1	1	124.58	19.63	0.0007
X_2	1	218.82	34.48	< 0.0001
X_3	1	99.62	15.70	0.0016
Residual	13	6.35		
Lack of fit	9	5.25	0.5954	0.7622
Pure error	4	8.82		
Total	16			

Note: df (Degree of freedom), a large F-value implies a large impact on the modeling profile; a p-value of less than 0.05 means that it affects modeling profile
